# Effects of opioid-free total intravenous anesthesia on postoperative nausea and vomiting after treatments of lower extremity wounds: protocol for a randomized double-blind crossover trial

**DOI:** 10.1186/s13741-023-00329-9

**Published:** 2023-07-14

**Authors:** Ya-juan Zhu, Dan Wang, Yu-qin Long, Long Qian, Hong Liu, Fu-hai Ji, Ke Peng

**Affiliations:** 1 Department of Anesthesiology, First Affiliated Hospital of Soochow University, Suzhou, 215006 Jiangsu China; 2 Institute of Anesthesiology, Soochow University, Suzhou, Jiangsu China; 3 Department of Anesthesiology, Guanyun People’s Hospital, Lianyungang, Jiangsu China; 4 Department of Anesthesiology and Pain Medicine, University of California Davis Health, Sacramento, CA USA

**Keywords:** Opioid-free anesthesia, Total intravenous anesthesia, Postoperative nausea and vomiting, Wound treatment surgery, Crossover design

## Abstract

**Background:**

Postoperative nausea and vomiting (PONV) are common after general anesthesia and surgery. This study aims to compare the effects of total intravenous opioid-free anesthesia (OFA) with conventional opioid-based anesthesia (OBA) on PONV in patients following treatments for wounds of lower extremities.

**Methods:**

This randomized, double-blind, crossover trial will include a total of 72 adult patients scheduled for at least two separate surgical treatments of lower extremity wounds under general anesthesia. Patients will be randomized to 1 of 2 anesthesia sequences of OFA and OBA. Patients in sequence 1 will receive OFA in the first treatment procedure and OBA in the second procedure, while patients in sequence 2 will receive the two anesthesia regimens in the reverse order. The washout period is at least 5 days. OFA will be delivered with intravenous esketamine, lidocaine, dexmedetomidine, and propofol. OBA will be delivered with intravenous sufentanil and propofol. The primary endpoint is the incidence of PONV within the first 48 h postoperatively. The secondary endpoints are the severity of PONV, antiemetic rescue therapy, postoperative pain scores, the worst pain, need for rescue analgesia, postoperative sedation, hypotension, bradycardia, hypertension, tachycardia, hypoxemia, psychotomimetic or dissociative effects, time to extubation, and length of postanesthesia care unit stay. Patients who complete two surgical procedures with designated anesthesia regimens will be included in the final analyses.

**Discussion:**

This crossover trial will determine whether total intravenous OFA reduces PONV in patients following treatments for lower extremity wounds. The results of this trial will also represent an important step to understand the benefits and possible risks of OFA in surgical patients.

**Trial registration:**

Chinese Clinical Trial Registry (ChiCTR2200061511).

**Supplementary Information:**

The online version contains supplementary material available at 10.1186/s13741-023-00329-9.

## Background

Postoperative nausea and vomiting (PONV) are frequent complications in patients who undergo general anesthesia and surgery. PONV not only leads to significant distress of patients but also is associated with an increased risk for postoperative morbidity and higher healthcare costs [1–4]. Preventing and reducing PONV are still challenging for practitioners in daily practice of perioperative care.

Opioid-free anesthesia (OFA) may alleviate PONV and facilitate recovery after surgery by reducing opioid-related adverse effects. The concept of OFA refers to a multimodal anesthesia regimen with the combined use several medications (α-2 agonists, N-methyl-D-aspartate antagonists, and local anesthetics) to replace opioids and provide intraoperative antinociception [5, 6]. Recent studies suggested that OFA may reduce the incidence and severity of PONV and decrease opioid consumption after surgery [7–9]. However, other studies argued that OFA did not reduce PONV and was associated with increased risks of bradycardia and sedation [10, 11]. Thus, whether the use of OFA could avoid opioid-related adverse events including PONV as well as the potential risks remains inconclusive.

We design this randomized crossover trial to test the primary hypothesis that our OFA regimen leads to a lower incidence of PONV, when compared to a conventional opioid-based anesthesia (OBA) regimen, in patients undergoing surgical treatments of lower extremity wounds. As for the secondary aims, we will explore the severity of PONV, postoperative pain outcomes, adverse effects, and recovery from anesthesia between the two groups. We report this protocol following the statement of Standard Protocol Items: Recommendations for Interventional Trials (SPIRIT) (Supplementary file 1) [12].

## Methods

### Ethics and registration

The Ethics Committee of The First Affiliated Hospital of Soochow University reviewed and approved this study protocol on April 28, 2022 (Approval No. 2022–108). After obtaining the ethic approval, we registered this trial at the Chinese Clinical Trial Registry (https://www.chictr.org.cn; identifier: ChiCTR2200061511; available at: https://www.chictr.org.cn/showprojEN.html?proj=172088). The implementation of this study will follow the Declaration of Helsinki. All patients will provide their written informed consent.

### Trial design

**Fig. 1:**
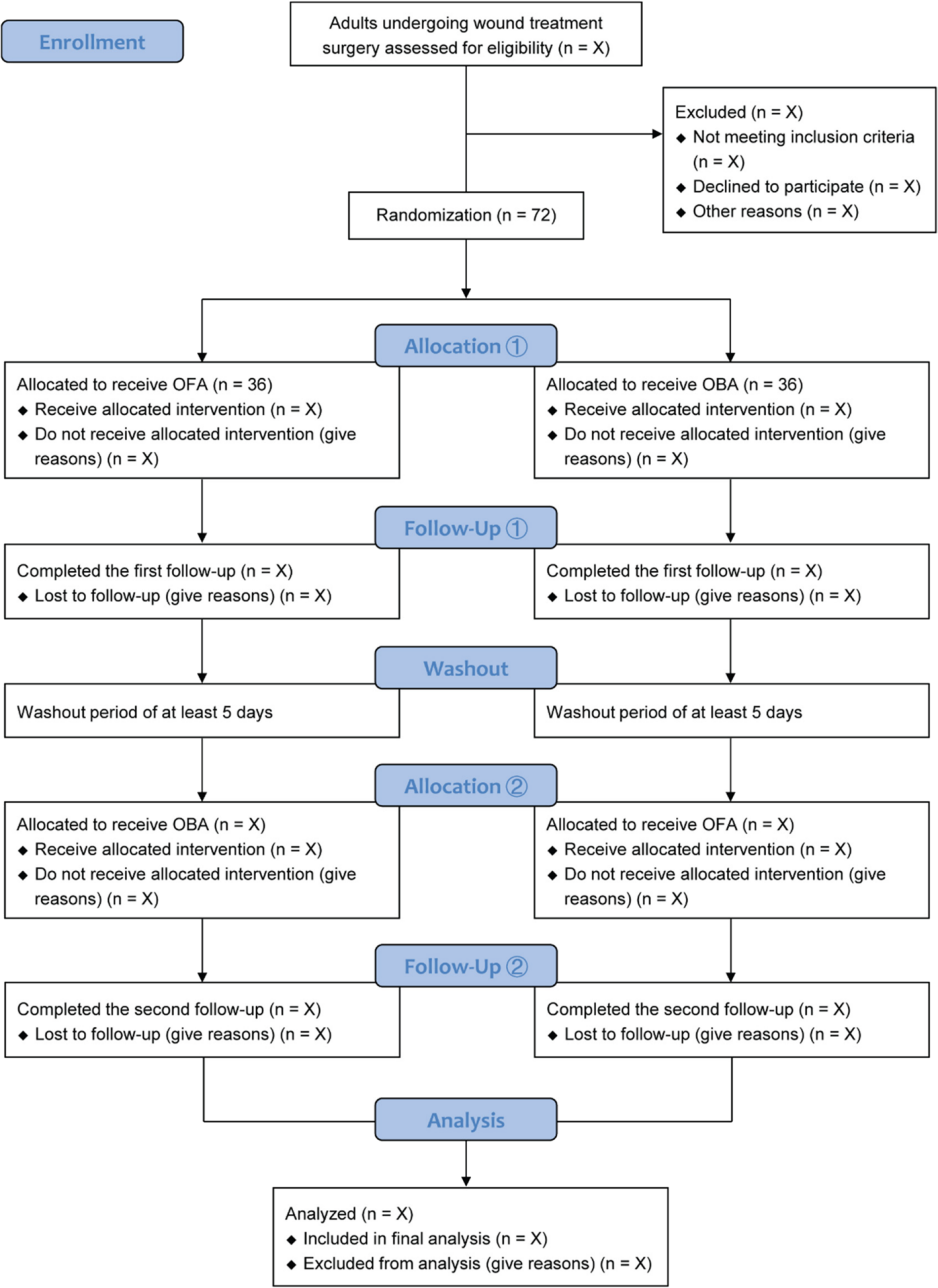
Study flowchart

This is an investigator-initiated, single-center, prospective, randomized, double-blind, controlled crossover trial. Based on the crossover design, a same patient will receive one of the two anesthesia regimens (OFA and OBA) in a random sequence during separate surgical procedures. This study is conducted at an academic teaching medical center (the First Affiliated Hospital of Soochow University, Suzhou, Jiangsu, China). Patient recruitment is ongoing. Figure 1 depicts the study flow diagram. Table 1 shows the schedule of patient enrollment, study interventions, and outcome measurements following the SPIRIT statement.

**Table 1:** Schedule of patient enrollment, study interventions, and outcome measurements

	**Study period**						
**Timepoint**	**Enrollment**	**Allocation**	**Post-allocation**				**Closeout**
	*Preoperative visit*	*Before surgery*	*During surgery*	*PACU*	*24 h after surgery*	*48 h after surgery*	*Hospital discharge*
**Enrollment**							
Inclusion criteria	×						
Exclusion criteria	×						
Written informed consent	×						
Baseline characteristics	×						
PONV risk score	×						
Randomization		×					
Allocation		×					
**Interventions**							
Sequence 1: OFA-OBA			×				
Sequence 2: OBA-OFA			×				
**Endpoint measures**							
Incidence of PONV				×	×	×	
Severity of PONV				×	×	×	
Antiemetic rescue therapy				×	×	×	
Postoperative pain scores				×	×	×	
The worst pain				×	×	×	
Need for rescue analgesia				×	×	×	
Level of sedation			×	×			
Hypotension			×	×			
Bradycardia			×	×			
Hypertension			×	×			
Tachycardia			×	×			
Hypoxemia				×	×	×	
Psychotomimetic/dissociative effects				×	×	×	
Time to extubation				×			
Length of PACU stay				×			
**Perioperative data**							
Patient satisfaction							×
In hospital major complications							×
Length of anesthesia				×			
Length of procedure				×			
Postoperative length of hospital stay							×

According to SPIRIT statement of defining standard protocol items for clinical trials

*OFA* opioid-free anesthesia, *OBA* opioid-based anesthesia, *PONV* postoperative nausea and vomiting, *PACU *postanesthesia care unit

### Inclusion criteria

The inclusion criteria are age ≥ 18 years old, American Society of Anesthesiologists physical status I–III, and having at least two separate treatments for lower extremity wounds under general anesthesia (an interval between the procedures ≥ 5 days). These treatment procedures include debridement and vacuum sealing drainage for wounds of lower extremities (such as diabetic foot ulcers, venous ulcers, pressure ulcers, wounds after orthopedic surgery, and traumatic wounds), excluding severely infected wounds or burn wounds. Skin grafts or flaps are used to cover the skin and soft-tissue defect if indicated.

### Exclusion criteria

The exclusion criteria are (1) unplanned or emergency surgery, (2) left ventricular ejection fraction < 40%, (3) second-degree or greater atrioventricular block, (4) sick sinus syndrome or severe bradycardia (heart rate [HR] < 50 beats/min), (5) severe liver or renal dysfunction (Child–Pugh grade C or renal replacement therapy), (6) epilepsy or seizures, (7) preoperative use of sedatives or analgesics, or (8) allergy to medications used in this study.

### Randomization and blinding

An independent investigational pharmacist generates the random numbers using a computer-generated process, with an allocation ratio of 1:1 and permuted block sizes of 2 and 4. The details of randomization will be kept in sealed opaque envelopes. Patients will be randomized to 1 of 2 treatment sequences: OFA-OBA (sequence 1) and OBA-OFA (sequence 2). The independent pharmacist formulates the study medications in the same fashion. These study medications (esketamine, lidocaine, and dexmedetomidine in the OFA regimen; sufentanil and normal saline in the OBA regimen) look identical. The anesthesia providers will be blinded to the group assignment. An independent trained investigator who is blinded to group allocation will assess the study outcome measures. The allocation and treatment codes will not be disclosed to patients or research personnel until the completion of final analysis.

### Study interventions

Patients in sequence 1 will receive OFA during the first treatment procedure and OBA during the second procedure. Patients in sequence 2 will receive OBA during the first treatment procedure and OFA during the second procedure. The washout period between the two treatments is at least 5 days. Table 2 shows the detailed administration of study medications.

**Table 2:** Details of study medications

	**OFA**	**OBA**
**Anesthesia induction**	Esketamine 0.2–0.4 mg/kg	Sufentanil 0.2–0.4 μg/kg
	Lidocaine 1 mg/kg	Normal saline
	Propofol 1.5–2.0 mg/kg	Propofol 1.5–2.0 mg/kg
**Anesthesia maintenance**	Dexmedetomidine 0.3–1.0 μg/kg/h	Normal saline infusion
	Esketamine 0.1 mg/kg boluses	Sufentanil 0.1 μg/kg boluses
	Propofol 2–10 mg/kg/h	Propofol 2–10 mg/kg/h

*OFA* opioid-free anesthesia, *OBA* opioid-based anesthesia

For patients receiving the OFA regimen, anesthesia will be induced with intravenous esketamine 0.2–0.4 mg/kg, lidocaine 1 mg/kg, and propofol 1.5–2.0 mg/kg. After that, anesthesia will be maintained with dexmedetomidine infusion 0.3–1.0 μg/kg/h, boluses of esketamine 0.1 mg/kg, and propofol infusion 2–10 mg/kg/h.

For patients receiving the OBA regimen, anesthesia will be induced with intravenous sufentanil 0.2–0.4 μg/kg, normal saline placebo, and propofol 1.5–2.0 mg/kg. After that, anesthesia will be maintained with normal saline infusion, boluses of sufentanil 0.1 μg/kg, and propofol infusion 2–10 mg/kg/h.

### Anesthetic care

In the preoperative waiting area, the baseline HR and mean blood pressure (MBP) will be recorded. In the operating room, patients will be monitored with electrocardiography, noninvasive blood pressure, pulse oximetry (SpO_2_), and bispectral index (BIS). General anesthesia will be induced and maintained according to the OBA or OFA regimen. After anesthesia induction, intravenous cisatracurium 0.15 mg/kg will be given for tracheal intubation. The lungs will be ventilated with tidal volume 8–10 ml/kg, 12–18 breaths/min, inspired oxygen fraction 50–80% in air, and positive end-expiratory pressure 5–8 cmH_2_O to maintain *SpO*_*2*_ ≥ 95% and end-tidal carbon dioxide 35–45 mmHg. Propofol infusion will be titrated to maintain the BIS values within 40–60. Intraoperative analgesia will be provided with dexmedetomidine and esketamine in the OFA regimen or sufentanil in the OBA regimen. Cisatracurium will be administered for tracheal intubation only, without additional doses during surgery. Neuromuscular blockade reversal agents (neostigmine or sugammadex) will not be routinely used. Local anesthesia (such as wound infiltration, regional nerves blocks, and epidural or spinal anesthesia) will not be performed in our patients. After surgery, patients will be extubated in a post-anesthesia care unit (PACU). A modified Aldrete score ≥ 9 indicates readiness for PACU discharge to surgical wards.

Prophylaxis of PONV includes intravenous dexamethasone 5 mg after anesthesia induction and intraoperative ondansetron 4 mg. At PACU discharge and 24 and 48 h postoperatively, the severity of PONV will be rated as none, mild (not compromising activities of daily living), moderate (sometimes compromising activities of daily living), or severe (inability to have activities of daily living or ≥ 3 vomits) [13, 14]. If patients experience severe PONV, antiemetic rescue therapy with additional ondansetron 4 mg will be given. For postoperative analgesia, patients will receive flurbiprofen axetil 50 mg at the end of surgery and every 12 h during the first 2 days postoperatively. Pain scores at 24 and 48 h postoperatively as well as the worst pain will be assessed using the numerical rating scale (*NRS*, 0–10; 0 = no pain, 10 = the most severe pain). If patients experience moderate-to-severe pain with NRS scores ≥ 4, rescue analgesia with intravenous nalbuphine or dezocine will be administered.

During surgery and in the PACU, hypotension (a decrease in *MBP* > 30% of baseline value), bradycardia (*HR* < 50 beats/min), hypertension (an increase in *MBP* > 30% of baseline value), and tachycardia (*HR* > 100 beats/min) will be treated using ephedrine, phenylephrine, atropine, esmolol, or urapidil at the discretion of the attending anesthesiologist. Sedation will be assessed in the PACU using the Richmond Agitation Sedation Scale, with a score of ≤  − 2 indicating postoperative sedation [10, 15]. Hypoxemia (*SpO*_*2*_ < 90% on room air) in the PACU and surgical ward will be managed with oxygen supplementation via a nasal catheter or mask ventilation if necessary.

### Study endpoints

The primary endpoint of this study is the incidence of PONV within the first 48 h postoperatively. The secondary endpoints are the severity of PONV, antiemetic rescue therapy, postoperative pain scores, the worst pain, need for rescue analgesia, postoperative sedation, hypotension, bradycardia, hypertension, tachycardia, hypoxemia, psychotomimetic or dissociative effects, time to extubation, and length of postanesthesia care unit stay.

### Perioperative non-endpoint data

Preoperative data include patients’ demographics and baseline characteristics. The risk of PONV will be assessed using the Apfel’s score, based on the number of risk factors (female sex, non-smoker, history of PONV or motion sickness, and postoperative opioid use) [16]. Postoperative non-endpoint data include patient satisfaction on the anesthesia regimens, inhospital major complications (myocardial infarction, cardiac arrest, coma, cerebrovascular accident, pulmonary embolism, acute renal failure, sepsis, failure to wean off ventilator, or death), length of anesthesia, length of procedure, and postoperative length of hospital stay.

### Data management

A trained independent investigator will collect the perioperative data in case report forms, and then data will be registered in the electronic database. After the completion of this trial, an independent statistician will access the deidentified data for statistical analyses according to the predefined statistical plan. The lead investigator is responsible for the accuracy and completeness of data. An independent data monitoring committee (DMC) will monitor the trial process and data management.

### Safety monitoring

As the administration and dosage of study medications in both anesthesia regimens are within the current clinical practice, we believe that serious adverse events should be rare. In case of rapid deterioration of patient status, the clinicians could discontinue the administration of study medications and request unmasking of group allocation. The DMC will conduct an ongoing review to safeguard patient safety. Throughout the study, any adverse events should be reported within 24 h to the DMC.

### Sample size

Previous studies showed that use of OFA, compared to OBA, reduced the incidence of PONV from 37.3 to 20% (an absolute reduction of 17.3%) following bariatric surgery [9] and from 33 to 13% (an absolute reduction of 20%) following laparoscopic cholecystectomy [8]. Our institutional data suggested that the incidence of PONV was approximately 30% in patients who had surgical wound treatment and OBA. We hypothesize that the OFA regimen would reduce the PONV incidence by 20% (i.e., from 30 to 10%) in this study. The power analysis suggests that 59 patients are needed in each group with *α* = 0.05 and power = 80%. Considering a possible dropout rate of 18%, we expand the sample size to 72 patients. They will be evaluated for both anesthetic techniques. The sample size calculation was performed using the PASS software (version 15.0.5, NCSS, LCC, Kaysville, UT, USA).

### Statistical analysis

Continuous variables will be presented as means (standard deviations) and medians (interquartile ranges) depending on data distribution. Categorical variables will be presented as numbers (percentages). The treatment effects will be analyzed using odds ratio or mean difference with 95% confidence intervals. A mixed linear model or logistic regression model will be used to test for treatment differences with sequence, period, and treatment as fixed effects and subject as a random effect. In addition, we will perform analyses adjusting for two covariates (Apfel’s PONV risk scores and length of procedure). Subgroup analyses will be conducted for the primary outcome according to sex and PONV risk scores. We have no plan of multiple testing correction for the secondary endpoints, and thus, these results should be interpreted as exploratory.

In this crossover trial, if a patient undergoes only one surgery after randomization, they will be regarded as dropouts. Patients who complete two surgical procedures with designated anesthesia regimens will be included in the final analyses. Statistical analyses will be performed using the SPSS (version 19.0; IBM SPSS, Chicago, IL, USA). Statistical significance is set at a 2-sided α level < 0.05 for all tests.

## Discussion

This randomized, double-blind, crossover trial will include 72 patients scheduled for two treatments of lower extremity wounds under general anesthesia. The primary objective is to determine whether the OFA regimen vs. the conventional OBA regimen reduces the incidence of PONV after the procedures. In addition, we will evaluate the severity of PONV, antiemetic use, postoperative pain, rescue analgesia, postoperative sedation and other adverse effects, and recovery from anesthesia between the two anesthesia groups. The implementation of this trial and the reporting of the results will follow the Consolidated Standards of Reporting Trials (CONSORT) guideline [17].

Bakan and colleagues evaluated the use of OFA with dexmedetomidine, lidocaine, and propofol in patients undergoing laparoscopic cholecystectomy, showing that OFA reduced postoperative fentanyl consumption within 2 days after surgery, the incidence of PONV, and the need for rescue antiemetics [8]. For patients undergoing bariatric surgery, the opioid-free total intravenous anesthesia with dexmedetomidine ketamine and propofol reduced the prevalence and severity of PONV in the postoperative period [9]. A recent study by Chen and colleagues showed that OFA with dexmedetomidine and esketamine reduced the incidence of PONV and improved the postoperative sleep quality after gynecological laparoscopic surgery under a protocol of enhanced recovery after surgery [18]. On the other hand, the possible drawbacks of OFA include increased risks of bradycardia, hypotension and sedation, a longer time to extubation, and a prolonged length of PACU stay [10, 11]. These adverse effects are often associated with a high dose of dexmedetomidine. In our study protocol, dexmedetomidine will be infused at a rate of 0.3–1.0 μg/kg/h without a loading dose, which may minimize the associated risks.

To provide intraoperative antinociception, OFA often consists of several medications including α-2 adrenergic receptor agonist (dexmedetomidine), N-methyl-D-aspartate antagonist (ketamine or esketamine), and local anesthetics [5, 6]. Local anesthesia (such as wound infiltration, regional nerves blocks, and epidural or spinal anesthesia) will not be performed in our patients, for the following reasons: (1) intravenous lidocaine is already used in the OFA regimen, (2) nerve blocks may increase the risk of nerve injury in patients with diabetic foot ulcers, and (3) general anesthesia is the routine practice for patients undergoing wound debridement and vacuum sealing drainage in our institution.

A major advantage of this trial is the crossover design (a two-sequence and two-period crossover AB/BA design). By using this study design, two separate anesthesia regimens will be provided to each patient during two different surgical periods, with the randomization of the sequence of treatments. Patients in the sequence 1 will receive OFA in the first procedure, followed by OBA in the second procedure after the effects of OFA have subsided. Patients in the sequence 2 will receive OBA first and then OFA. We designate a washout period of at least 5 days to avoid possible carryover effect of the first anesthesia treatment. Therefore, each patient serves as their own control, and the therapeutic effects of OFA vs. OBA will be analyzed within a patient (i.e., a within-subject comparison). This could avoid the imbalance of allocation in a parallel trial (i.e., a between-subject comparison). Moreover, the crossover design improves the statistical power and efficiency [19].

To our knowledge, this is the first randomized, double-blind, controlled crossover trial to evaluate the effects of OFA vs. OBA on PONV following anesthesia and surgery. This study also has some limitations. First, the definitions and components of OFA vary in the existing literature [8–11, 20–22]. The regimen of OFA in our study is not definitive, and the best approach to deliver OFA requires further investigation. Next, patients will undergo two or more surgical procedures for wound treatment in this crossover trial. The types of two procedures may be different, which may confound the study results. Last, this is a single-center trial with a small sample size, and more studies are needed to test the generalizability of our results.

In conclusion, this crossover trial will determine the effects of OFA regimen, as compared to the OBA regimen, on the incidence of severity of PONV, postoperative pain, and adverse events after treatments for lower extremity wounds. Our findings will represent a step toward better understanding of the benefits and potential risks of OFA in patients undergoing anesthesia and surgery.

## Supplementary Information

**Additional file 1.** SPIRIT 2013 Checklist: Recommended items to address in a clinical trial protocol and related documents*.

## Data Availability

All data relevant to the study protocol is included as part of this manuscript. The prospective listing of the study on the Chinese Clinical Trial Registry can be found at http://www.chictr.org.cn/showproj.aspx?proj=172088.
